# Association between Serum Interleukin-6 Concentrations and Mortality in Older Adults: The Rancho Bernardo Study

**DOI:** 10.1371/journal.pone.0034218

**Published:** 2012-04-13

**Authors:** Jeffrey K. Lee, Ricki Bettencourt, David Brenner, Thuy-Anh Le, Elizabeth Barrett-Connor, Rohit Loomba

**Affiliations:** 1 Department of Medicine, University of California San Diego, La Jolla, California, United States of America; 2 Division of Gastroenterology, University of California San Diego, La Jolla, California, United States of America; 3 Division of Epidemiology, Department of Family and Preventive Medicine, University of California San Diego, La Jolla, California, United States of America; German Diabetes Center-, Leibniz Center for Diabetes Research at Heinrich Heine University Duesseldorf, Germany

## Abstract

**Background:**

Interleukin-6 (IL-6) may have a protective role in acute liver disease but a detrimental effect in chronic liver disease. It is unknown whether IL-6 is associated with risk of liver-related mortality in humans.

**Aims:**

To determine if IL-6 is associated with an increased risk of all-cause, cardiovascular disease (CVD), cancer, and liver-related mortality.

**Methods:**

A prospective cohort study included 1843 participants who attended a research visit in 1984–87. Multiple covariates were ascertained including serum IL-6. Multivariable-adjusted Cox proportional hazards regression analyses were used to examine the association between serum IL-6 as a continuous (log transformed) variable with all-cause, CVD, cancer, and liver-related mortality. Patients with prevalent CVD, cancer and liver disease were excluded for cause-specific mortality.

**Results:**

The mean (± standard deviation) age and body-mass-index (BMI) of participants was 68 (±10.6) years and 25 (±3.7) Kg/m^2^, respectively. During the 25,802 person-years of follow-up, the cumulative all-cause, CVD, cancer, and liver-related mortality were 53.1% (N = 978), 25.5%, 11.3%, and 1.3%, respectively. The median (±IQR) length of follow-up was 15.3±10.6 years. In multivariable analyses, adjusted for age, sex, alcohol, BMI, diabetes, hypertension, total cholesterol, HDL, and smoking, one-SD increment in log-transformed serum IL-6 was associated with increased risk of all-cause, CVD, cancer, and liver-related mortality, with hazard ratios of 1.48 (95% CI, 1.33–1.64), 1.38 (95% CI, 1.16–1.65), 1.35 (95% CI, 1.02–1.79), and 1.88 (95% CI, 0.97–3.67), respectively. CRP adjustment attenuated the effects but the association between IL-6 and all-cause and CVD mortality remained statistically significant, independent of CRP levels.

**Conclusions:**

In community-dwelling older adults, serum IL-6 is associated with all-cause, CVD, cancer, and liver-related mortality.

## Introduction

Interleukin-6 (IL-6) is a pleiotropic cytokine that has a differential effect on tissue growth, repair, and regeneration [1]–[3]. It may be essential for tissue regeneration in the acute setting, but under certain conditions, prolonged exposure to IL-6 may promote carcinogenesis [2]. Acute versus chronic exposure to IL-6 may have opposing effect on normal versus altered growth, development and cell death [3]. During acute inflammation, IL-6 promotes the expansion and activation of T cells, initiates B cell differentiation, regulates the liver production of acute phase reactants such as C-reactive protein (CRP), and stimulates the hypothalamic-pituitary-adrenal axis, which may be protective in an acute setting [4]. However, prolonged exposure to IL-6 may lead to activation of apoptosis, cell death and lower the threshold for liver injury [3] Recent studies have shown that high serum IL-6 levels increase the risk of cardiovascular disease (CVD) and major coronary events [5]–[7]. In addition, high IL-6 mRNA expression is found in atherosclerotic arteries of patients undergoing heart transplantation and atherectomy [8]. In liver disease, elevated IL-6 levels are seen in alcoholic cirrhosis, chronic hepatitis B, primary biliary cirrhosis, hepatitis C (HCV) cirrhosis, and hepatocellular carcinoma (HCC) [9], [10]. IL-6 expression also correlates with disease severity in non-alcoholic fatty liver disease (NAFLD) and is higher in nonalcoholic steatohepatitis (NASH) than steatosis alone [11]. These findings suggest that IL-6 is important in the pathogenesis and progression of cardiovascular as well as chronic liver disease. Despite improved understanding of IL-6′s role in inflammation and chronic disease, there are limited data on the effect of IL-6 on CVD, cancer, and liver-related mortality in a large population-based cohort. In this study, we investigate the predictive value of IL-6 levels for all-cause, CVD, cancer, and liver-related mortality using a large, prospective cohort of older men and women in Southern California. We hypothesized that 1.) Serum IL-6 is associated with an increased risk of all-cause, CVD, cancer, and liver-related mortality in older adults and 2.) The association between serum IL-6 and mortality is independent of CRP levels.

## Methods

### Study Design and Population

The Rancho Bernardo Study (RBS) is a prospective cohort study of older adults from a suburban Southern California community. A total of 2480 adult residents from the original cohort attended a clinical research examination between 1984 and 1987. This visit included a standardized questionnaire on medical and medication history, alcohol consumption, smoking status, and physical activity. Detailed information on the prevalence of CVD, and other major chronic diseases along with objective measures of disease severity were obtained. The details of the cohort, selection criteria, and purpose of the RBS have been published previously [12], [13].

### Ethics Statement

The institutional review board of the University of California, San Diego approved the study protocol and a written informed consent was obtained from all participants.

### Exposure: Serum IL-6

IL-6 was measured in fasting serum samples using a high sensitivity (0.094 pg/ml) commercial Elisa (Quantikine HS, human IL-6 immunoassay; R&D Systems, Minneapolis, MN).

### Mortality Follow-Up

Mortality status was determined by annual mailed questionnaires until December 31, 2005. Death certificates were obtained from all decedents in this cohort; cause of death was classified by a nosologist using the International Classification of Diseases (ICD), Ninth Revision. Cancer deaths, CVD deaths, and deaths from all causes included codes 140–239 (excluding 210–229), 390–459, and 0–999 respectively. Liver deaths included underlying or associated cause of death by codes 70.2–70.9, 155, 275.0–275.1, 571–573.

### Covariate Assessment

Information for covariate analyses included age, sex, body mass index (BMI), fasting serum total cholesterol and high-density lipoprotein (HDL), fasting plasma glucose, measured blood pressure, smoking status, alcohol use, and CRP levels. CRP was measured using an automated, high-sensitivity immunonephelometry method (sensitivity of 0.2 mg/liter, Dade Behring, Inc., Deerfield, IL). Participants had hypertension if they had an average systolic blood pressure ≥140 mm Hg, diastolic blood pressure ≥90, or were receiving anti-hypertensive medications. Diabetes mellitus was defined as fasting plasma glucose ≥126 mg/dl or treatment with either insulin or an oral hypoglycemic medication. A current smoker or anyone who smoked 10 cigarettes or more in the last year was classified as a smoker. Alcohol use history including frequency, type, and quantity of alcoholic beverages consumed in the past week was obtained. Alcohol was considered as the number of alcoholic drinks (beer, wines, or spirits) taken in a day, with one drink equivalent defined as either 12-ounces of beer or 8-ounces of malt liquor or 5-ounces of wine or 1.5-ounces of a “shot” of 80-proof distilled spirits or liquor (e.g., gin, rum, vodka, or whiskey) according to the Centers for Disease Control. Non-drinkers were defined as no alcohol use in the last year.

### Statistical Analysis

Descriptive statistics were used to compare the baseline characteristics across tertiles of IL-6. Chi-square test was used to examine the significance for trends from models with tertiles of IL-6 concentration entered as an ordinal variable. We used the Cox proportional hazards regression analysis to examine the hazards ratio of all-cause, CVD, cancer, and liver-related mortality associated with serum IL-6 levels as a continuous variable (IL-6 was log transformed to fulfill the conditions of normality for these analyses) and also IL-6 divided into tertiles, with tertile 3 with highest IL-6 and tertile 1 with lowest IL-6. We also performed a hierarchical adjustment and examined the following models: 1) age-sex adjusted, 2) multivariate model adjusted for age, sex, alcohol, BMI, diabetes mellitus, systolic blood pressure, total cholesterol, HDL, smoking, 3) Model 2 plus baseline CRP. IL-6 correlated significantly with CRP (Spearman correlation r^2^ = 0.49, p<0.001). BMI categories were as follows: BMI <18.5 kg/m^2^, (normal BMI) 18.5–25 kg/m^2^ as referent, BMI 25–30 kg/m^2^, and BMI ≥30 kg/m^2^. Alcohol use was categorized as non-drinker, less than median number of drinks, and greater than median number of drinks as shown in table 1. Lastly, we performed all models examining the association between serum IL-6 and CVD, cancer, and liver-related mortality by excluding participants with prevalent CVD, cancer, or liver disease at baseline. Information on prevalent CVD (including angina, myocardial infarction, cardiac revascularization, stroke or transient ischemic attack, carotid surgery, peripheral arterial surgery, and intermittent claudication as previously reported) [14], cancer, and liver disease were obtained by self-report, review of hospital admission records, and questionnaires during baseline and annual follow-up mailers. We also performed *Wald* test of interaction to examine the interaction between IL-6 and age/sex for all the mortality outcomes. A two-sided P-value <0.05 was considered statistically significant. SAS version 9.1 (SAS Institude, Cary, NC) was used for all analyses.

**Table 1:** Baseline characteristics of participants based on tertiles of IL-6 in the Rancho Bernardo Study cohort.

	N	Total Sample	Tertile 1 (N = 615)	Tertile 2 (N = 615)	Tertile 3 (N = 613)	P-Value (trend)
**IL-6 Mean ± SD (pg/ml)**			1.23 ± 0.34 pg/ml	2.31 ± 0.36 pg/ml	5.84 ± 3.16 pg/ml	
**IL-6 Median ± IQR (pg/ml)**	1843	2.27 ± 2.16 pg/ml	1.29 ± 0.48 pg/ml	2.27 ± 0.62 pg/ml	4.53 ± 3.25 pg/ml	
**Age, years (SD)**	1843	68.4 (10.6)	64.0 (10.8)	69.5 (9.7)	71.5 (9.8)	**<.0001**
**Women, n (%)**	1843	1029(55.8)	394 (64.1)	317 (51.5)	318 (51.9)	**<.0001**
**Body mass index (kg/m2)**	1843					
Mean, SD		25.0 (3.7)	24.2 (3.2)	25.3 (3.6)	25.5 (4.0)	**<.0001**
<18.5, n (%)		31 (1.7)	8 (1.3)	12 (2.0)	11 (1.8)	**<.0001** *
18.5 −<25, n (%)		965 (52.3)	378 (61.4)	304 (49.4)	283 (46.2)	
25 −<30, n (%)		691 (37.5)	199 (32.4)	242 (39.3)	250 (40.8)	
≥30, n (%)		156 (8.5)	30 (4.9)	57 (9.3)	69 (11.2)	
**Alcohol use, n (%)**	1843					
None		678 (36.8)	224 (36.4)	210 (34.1)	244 (39.8)	**.0026** *
< median drinks/day **		552 (29.9)	192 (31.2)	212 (34.5)	148 (24.1)	
≥ median drinks/day		613 (33.3)	199 (32.4)	193 (31.4)	221 (36.1)	
**SBP (SD)**	1841	137.4 (21.9)	130.4 (20.2)	137.8 (20.6)	144.1 (22.7)	**<.0001**
**Hypertension, n (%)**	1843	1314 (71.3)	354 (57.6)	458 (74.5)	502 (81.9)	**<.0001**
**Lipid levels (SD)**						
Total cholesterol (mg/dl)	1843	220.2 (39.8)	224.3 (37.4)	220.2 (40.5)	216.0 (41.0)	**.0003**
HDL (mg/dl)	1843	61.4 (18.7)	66.0 (18.5)	60.7 (18.0)	57.5 (18.6)	**<.0001**
Trig (mg/dl), geo mean (95% CI)	1843	103.0 (100.4–105.6)	91.5 (87.8–95.4)	105.3 (101.1–109.7)	113.3 (108.1–118.8)	**<.0001**
Total/HDL ratio	1843	3.9 (1.3)	3.6 (1.1)	3.9 (1.3)	4.1 (1.5)	**<.0001**
**FPG mg/dl (SD)**	1843	100.7 (20.3)	97.7 (17.2)	101.3 (20.0)	103.0 (23.1)	**<.0001**
**Diabetes, n (%)**	1843	253 (13.7)	43 (7.0)	93 (15.1)	117 (19.1)	**<.0001**
**Current smoker, n (%)**	1843	280 (15.2)	73 (11.9)	100 (16.3)	107 (17.5)	**.0064**
**CRP, geo mean (95% CI)**	1792	1.8 (1.7–1.9)	1.0 (0.9–1.1)	1.7 (1.6–1.9)	3.6 (3.3–3.9)	**<.0001**

Abbreviations: SD; standard deviation, SBP; systolic blood pressure, HDL; high density lipoprotein, FPG; fasting plasma glucose, CRP; C-reactive protein, IL-6; interleukin-6,

* Chi-square for association – can't compute trend test, more than 2 rows.

** Median number of drinks/day among drinkers was 1.1.

## Results

### Description of the Population

Baseline demographic, clinical, and laboratory characteristics of the 1843 (55.8% women) participants are shown in Table 1. The mean (± standard deviation, SD) age and BMI was 68.4 (±10.6) years and 25.0(±3.7) kg/m^2^, respectively. Participants with high IL-6 levels (tertile 3 = 3.02–16.45 pg/ml) were significantly older, more male, had higher BMI, SBP, triglycerides, fasting plasma glucose, CRP levels, and were more likely to be active smokers, diabetics as compared to participants with low IL-6 levels (tertile 1 = 0.10–1.74 pg/ml). Participants with low IL-6 levels had higher total cholesterol and HDL compared to those with high IL-6 levels.

### Mortality Follow-Up

During the 25,802 person-years of follow-up, the cumulative all-cause, CVD, cancer, and liver-related mortality were (N = 978) 53.1%, 25.5%, 11.3%, and 1.3%, respectively. The median (± IQR) length of follow-up was 15.3±10.6 years. There were 23 deaths due to liver disease as an underlying cause of death with only one death attributed to hepatocellular carcinoma (HCC).

### Serum IL-6 as a Continuous Variable and Mortality Risk

#### All-cause Mortality

One standard deviation increment in serum IL-6 was associated with 90% excess risk of all-cause mortality (Table 2). In order to put the logarithmic scale into clinical perspective, here, we provide an example to explain the risk difference in IL-6 levels in pg/ml; the risk of mortality increased by 90% when the IL-6 levels increased from 2.29 pg/ml to 4.56 pg/ml (difference of 2.27 pg/ml, which is equivalent to the standard deviation at the median). These results remained statistically significant in age-sex adjusted, multivariable adjusted, and multivariable with CRP adjusted models

**Table 2:** The hazards associated with increased IL-6 and risk of all-cause, cardiovascular, cancer, and liver-related mortality in older adults excluding individuals with prevalent CVD, cancer, and liver disease over 15 years of median follow-up.

	N	Natural log-transformed IL-6 HR (95% CI)	P-value
**All-cause Mortality**	No. of total deaths/total participants		
Unadjusted	978/1843	**1.90 (1.74–2.07)**	**<.0001**
Age-sex adjusted	978/1843	**1.53 (1.39–1.68)**	**<.0001**
Multivariable adjusted	976/1841	**1.48 (1.33–1.64)**	**<.0001**
Multivariable+CRP adjusted	946/1790	**1.39 (1.23–1.56)**	**<.0001**
**CVD Mortality (exclude individuals with prevalent CVD; N = 323 )**	No. of CVD deaths/total participants		
Unadjusted	329/1520	**1.89 (1.64–2.19)**	**<.0001**
Age-sex adjusted	329/1520	**1.46 (1.23–1.72)**	**<.0001**
Multivariable adjusted	329/1518	**1.38 (1.16–1.65)**	**.0004**
Multivariable+CRP adjusted	319/1476	**1.41 (1.15–1.72)**	**.0009**
**Cancer Mortality (exclude individuals with prevalent cancers; N = 447)**	No. of cancer deaths/total eligible participants		
Unadjusted	134/1396	**1.74 (1.37–2.20)**	**<.0001**
Age-sex adjusted	134/1396	**1.51 (1.17–1.96)**	**.0017**
Multivariable adjusted	134/1395	**1.35 (1.02–1.79)**	**.0343**
Multivariable+CRP adjusted	130/1359	0.99 (0.71–1.38)	.9400
**Liver Disease Mortality (exclude individuals with liver disease; N = 52)**	No. of liver deaths/total eligible participants		
Unadjusted	20/1691	**2.28 (1.27–4.10)**	**.0058**
Age-sex adjusted	20/1691	**2.10 (1.11–3.96)**	**.0218**
Multivariable adjusted	20/1689	1.88 (0.97–3.67)	.0633
Multivariable+CRP adjusted	20/1641	1.80 (0.84–3.85)	.1277

HR; hazards ratio, CI; confidence interval.

statistically significant, a two-tailed p-value of less than 0.05 (bold).

Multivariate model includes: age, sex, alcohol, BMI, diabetes mellitus, systolic blood pressure, total chol, HDL, smoking.

Multivariate model+CRP includes: age, sex, alcohol, BMI, diabetes mellitus, systolic blood pressure, total chol, HDL, smoking, CRP.

#### CVD Mortality

Serum IL-6 was associated with a nearly two-fold increased risk of CVD mortality. After exclusion of participants with known CVD at baseline, serum IL-6 continued to be a strongly associated with CVD mortality in the unadjusted, age-sex adjusted, multivariable adjusted, and multivariable with CRP adjusted models (Table 2).

#### Cancer Mortality

Serum IL-6 was associated with 74% excess risk of cancer mortality after excluding participants with cancer at baseline (Table 2). Results remained consistent serum IL-6 associated with cancer mortality in unadjusted, age-sex adjusted, and multivariable adjusted models (Table 2). However, adjustment by CRP attenuated the association between IL-6 and cancer mortality.

#### Liver Mortality

In unadjusted analysis, one log increment in serum IL-6 was associated with a two-fold increased risk (HR 2.25, 95% CI 1.30–3.90) of liver-related mortality (data not shown). These results persisted after adjusting for variables previously shown to increase mortality in this population. CRP attenuated the association between serum IL-6 and liver mortality. When prevalent liver disease was excluded, serum IL-6 remained significantly associated with liver-related mortality in unadjusted (HR 2.28, 95% CI 1.27–4.10) and age-sex adjusted (HR 2.10, 95% CI 1.11–3.96) models only (Table 2).

### Serum IL-6 Stratified Into Tertiles and Mortality Risk:

#### All-cause Mortality

When stratified into tertiles, high IL-6, tertile 3, was significantly associated with all-cause mortality in unadjusted (HR 2.99, 95% CI 2.54–3.53), age-sex adjusted, multivariable adjusted, and multivariable with CRP adjusted models when compared to the two lowest combined or the lowest IL-6 levels in tertile 1 (Table 3). In all models, serum IL-6 was strongly associated with all-cause mortality independent of CRP and all other covariates.

**Table 3:** Hazard ratios of all-cause, cardiovascular, cancer, and liver mortality stratified by IL-6 tertiles over 15 years of median follow-up (excluding individuals with prevalent CVD, cancer, and liver disease).

	N	Tertile 1	Tertile 2 HR (95% CI)	Tertile 3 HR (95% CI)	p for linear trend
**All-cause Mortality**					
No. of deaths	978	214	351	413	
Unadjusted	1843	Referent	**2.02 (1.71–2.40)**	**2.99 (2.54–3.53)**	**< .0001**
Age-sex adjusted	1843	Referent	**1.34 (1.13–1.59)**	**1.80 (1.52–2.13)**	**< .0001**
Multivariable adjusted	1841	Referent	**1.31 (1.10–1.57)**	**1.71 (1.44–2.05)**	**< .0001**
Multivariable+CRP adjusted	1790	Referent	**1.26 (1.06–1.51)**	**1.53 (1.26–1.85)**	**< .0001**
**CVD Mortality 323 excluded)**					
No. of deaths	329	83	103	143	
Unadjusted	1520	Referent	**1.69 (1.26–2.25)**	**3.04 (2.31–3.98)**	**< .0001**
Age-sex adjusted	1520	Referent	1.09 (0.82–1.47)	**1.65 (1.25–2.18)**	**.0002**
Multivariable adjusted	1518	Referent	1.06 (0.79–1.43)	**1.53 (1.14–2.05)**	**.0030**
Multivariable+CRP adjusted	1476	Referent	1.09 (0.80–1.48)	**1.52 (1.10–2.10)**	**.0077**
**Cancer Mortality (447 excluded)**					
No. of deaths	129	36	41	52	
Unadjusted	1396	Referent	1.44 (0.92–2.26)	**2.26 (1.47–3.46)**	**.0002**
Age-sex adjusted	1396	Referent	1.03 (0.65–1.63)	**1.62 (1.04–2.52)**	**.0256**
Multivariable adjusted	1395	Referent	0.86 (0.54–1.37)	1.28 (0.81–2.04)	.2309
Multivariable+CRP adjusted	1359	Referent	0.72 (0.45–1.16)	0.80 (0.47–1.36)	.4469
**Liver Disease Mortality (52 excluded)**					
No. of deaths	20	6	5	9	
Unadjusted	1691	Referent	1.03 (0.31–3.39)	2.21 (0.78–6.27)	.1314
Age-sex adjusted	1691	Referent	0.74 (0.22–2.50)	1.55 (0.52–4.58)	.3786
Multivariable adjusted	1689	Referent	0.64 (0.19–2.19)	1.20(0.38–3.78)	.6677
Multivariable+CRP adjusted	1641	Referent	0.58 (0.17–2.01)	0.94 (0.26–3.34)	.9927

Statistically significant, when 95% CI does not include 1 (bold).

Multivariate model includes: age, sex, alcohol, BMI, diabetes mellitus, systolic blood pressure, total chol, HDL, smoking.

Multivariate model+CRP includes: age, sex, alcohol, BMI, diabetes mellitus, systolic blood pressure, total chol, HDL, smoking, CRP.

#### CVD Mortality

The association between IL-6 and CVD mortality remained consistent between serum IL-6 stratified into tertiles and CVD mortality in the unadjusted model with tertile 3 (HR 3.04, 95% CI 2.31–3.98) and tertile 2 (HR 1.69, 95% CI 1.26–2.25) versus tertile 1 (P value for trend <0.0001, Table 3). In all models, serum IL-6 was strongly associated with incident CVD mortality independent of CRP and other covariates.

#### Cancer Mortality

In separate analyses using tertiles, high IL-6 levels was associated with a statistically significant increased risk of cancer mortality in unadjusted, and age-sex adjusted models (Table 3). Multivariate-adjusted models were not significant the association (Table 3).

#### Liver Mortality

When analyzed as tertiles, high IL-6 levels was associated with increased risk of liver mortality but failed to reach significance, likely reflecting limited power with only 23 (3 excluded due to prevalent liver disease at baseline) persons who had liver-related mortality on their death certificates (Table 3).

Sensitivity Analysis: *Wald* test for interaction was utilized to assess interaction between serum IL-6 levels and age (below and above the median age of 70 years at baseline), and sex. There was no interaction between serum Il-6 and age (interaction p-value of 0.2). However, the interaction between IL-6 and sex was significant (interaction p-value <0.002) but the hazards of mortality did not differ in men versus women (p-value 0.5) consistent with assumptions that serum Il-6 levels are higher in men than women. There were no significant interactions between any of the outcomes and sex/age (data not shown).

## Discussion

### Main Findings

In these community-dwelling older men and women with a median follow-up of 15.3±10.6 years, increased serum IL-6 levels were associated with an increased risk of all-cause, CVD, cancer, and liver-related mortality. After adjusting for age, sex, BMI, total cholesterol, HDL, serum glucose, systolic blood pressure, smoking status, and alcohol use, serum IL-6 remained independent risk factor of all-cause, CVD, cancer, and liver-related mortality. When CRP was added to our multivariable analyses, serum IL-6 was only associated with all-cause and CVD mortality, suggesting a novel hypothesis that the association between IL-6 and cancer and liver mortality is perhaps mediated via CRP-dependent pathways whereas the association between IL-6 and CVD mortality is not entirely dependent upon CRP. This remains to be tested in future studies.

### Association of IL-6 and mortality in previously published literature

Several observational studies have examined the association between serum IL-6 and mortality in older adults with conflicting results. The Iowa 65+ Rural Health Study showed that high IL-6 levels were associated with a two-fold greater risk of all-cause mortality in 1,293 elderly men and women [15]. In contrast, Arai et al. reported that serum IL-6 was not associated with all-cause mortality in 285 subjects with a mean age of 101.5 [16]. The Vitality 90+ Study showed that serum IL-6 was not associated with all-cause mortality [17]. More recently, the MEMO Study of 385 adults showed that serum IL-6 was an independent risk factor of all-cause mortality in elderly men [18]. These conflicting findings may be explained by the small samples size in several of these studies, short follow-up periods, and higher age of the participants suggesting survivor bias. Our population-based cohort study enrolled a larger number of participants, had a longer mean follow-up period, and a younger population compared to the studies above. As a result, we were able to show a clear association between serum IL-6 and all-cause mortality.

Although the association between IL-6, CVD, and CVD risk factors such as diabetes has been recognized in previous cohorts, few studies have examined the association between serum IL-6 and CVD mortality. Harris et al. reported that high IL-6 levels were not an independent risk factor of CVD mortality in 1,293 elderly men and women [15]. In contrast, the Women's Health and Aging Study found that high IL-6 levels were an independent risk factor of CVD mortality among older women [19]. A study of 403 elderly men supported that serum IL-6 was an independent risk factor of CVD mortality [20]. However, both studies used gender-specific recruiting strategies which may limit their findings [19], [20]. In our study, we demonstrated that serum IL-6 is an independent risk factor of CVD mortality in both older men and women. These results are consistent with published data and support the potential role of inflammation in the progression of CVD.

IL-6 has been shown to be an independent prognostic marker in cancer-specific mortality, including diffuse large-cell lymphoma, metastatic hormone-refractory prostate cancer, and colorectal cancer [21]–[23]. More recently, the PROSPER study showed that high IL-6 levels was associated with an increased risk for cancer incidence and cancer-related mortality in 5,804 elderly participants [24]. However, this study was limited by a short mean follow-up period of 3.2 years and the inclusion criteria of preexisting vascular disease or having an increased risk for such a disease (i.e. smoking, hypertension, diabetes) [24]. In our study, we demonstrated that IL-6 is an independent risk factor of cancer-related mortality in older adults with a longer mean follow-up period and fewer percentages of diabetics, smokers, and obese participants.

However, no previous epidemiological studies have investigated the association between serum IL-6 and liver-related mortality in a population-based cohort study. To the best of our knowledge, this is the first study to show that serum IL-6 is associated with an increased risk of liver-related in a community-dwelling cohort of older men and women. Future investigations are needed to fully elucidate and confirm our findings.

### Potential Mechanism

The mechanism underlying the increased risk of liver mortality with serum IL-6 is unclear. In rodents, IL-6 is a pro-growth factor required for hepatic regeneration and survival after partial hepatectomy [25], [26]. Sun et al. showed that in vitro IL-6 treatment reduced mortality associated with fatty liver transplants from alcohol-fed rats [27]. In contrast to its protective effect, several human and animal studies have demonstrated that IL-6 is elevated in alcoholic liver disease, NAFLD, NASH, and cirrhosis [9], [11], [28], [29]. Moreover, IL-6 expression is increased in both Kupffer cells and hepatocytes with levels correlating to both the stage of fibrosis and the inflammatory activity in humans with NASH [11]. Additionally, there is convincing evidence linking IL-6 to HCC [30], [31]. IL-6 induced STAT3 activation via Janus Kinases (JAK) has been implicated in HCC [32], [33].

These findings together suggest that IL-6 is needed acutely for hepatic protection, regeneration, and survival but chronic exposure may sensitize the liver to injury and apoptotic cell death. Although our study cannot test this hypothesis, we believe that chronic exposure to excess IL-6 levels in the liver may cause persistent liver damage leading to progressive liver disease, further increasing the risk of HCC and liver-related mortality. We further propose that liver and cancer mortality are mediated via CRP dependent pathways but CVD mortality may not be dependent upon CRP. Further research is needed to evaluate the mechanisms of association between IL-6 and cause-specific mortalities.

### Limitations and strengths of our Study

First, the Rancho Bernardo cohort consists of relatively healthy, middle to upper-middle class Caucasians, which limits the generalizability of our study to other more diverse groups. However, this was typical of U.S. suburbs in the 1970s when this community was established. Second, there were few liver-related deaths based on the death certificates, limiting power to detect an association between tertiles of IL-6 levels and liver-related mortality. The literature suggests that using underlying cause of death alone may grossly underestimate liver mortality [34], [35]. Third, viral hepatitis status was not ascertained; however among persons 60 years of age and older, the U.S. prevalence of hepatitis C was 0.9% between 1999 through 2002 [36].

Despite these limitations, the major strengths of our study include the large sample size, inclusion of both men and women, prospective study design, detailed evaluation of participants at baseline, well-characterized covariates, and nearly complete follow-up for vital status.

### Conclusions

In summary, serum IL-6 was a risk factor of all-cause, CVD, cancer, and liver-related mortality in community-dwelling older adults. Serum IL-6 was associated with an increased risk of all-cause and CVD mortality, independent of CRP levels. Our findings support the important role of chronic inflammation in the pathogenesis and progression of the two most common causes of death and advanced liver disease. Additional studies are needed to determine whether IL-6 may be utilized as a novel prognostic biomarker for earlier diagnosis and treatment in the future.
